# Telomere-dependent and telomere-independent origins of endogenous DNA damage in tumor cells

**DOI:** 10.18632/aging.100019

**Published:** 2009-02-04

**Authors:** Asako J. Nakamura, Christophe E. Redon, William M. Bonner, Olga A. Sedelnikova

**Affiliations:** Laboratory of Molecular Pharmacology, Center for Cancer Research, National Cancer Institute, National Institutes of Health, Bethesda, MD 20892, USA

**Keywords:** DNA damage, double-strand breaks, γ-H2AX, metaphase, telomeres, tumors

## Abstract

Human tumors and cultured cells contain elevated levels of endogenous DNA damage resulting from telomere dysfunction, replication and transcription errors, reactive oxygen species, and genome instability. However, the contribution of telomere-associated versus telomere-independent endogenous DNA lesions to this damage has never been examined. In this study, we characterized the relative amounts of these two types of DNA damage in five tumor cell lines by noting whether γ-H2AX foci, generally considered to mark DNA double-strand breaks (DSBs), were on chromosome arms or at chromosome ends. We found that while the numbers of non-telomeric DSBs were remarkably similar in these cultures, considerable variation was detected in the level of telomeric damage. The distinct heterogeneity in the numbers of γ-H2AX foci in these tumor cell lines was found to be due to foci associated with uncapped telomeres, and the amount of total telomeric damage also appeared to inversely correlate with the telomerase activity present in these cells. These results indicate that damaged telomeres are the major factor accounting for the variability in the amount of DNA DSB damage in tumor cells. This characterization of DNA damage in tumor cells helps clarify the contribution of non-telomeric DSBs and damaged telomeres to major genomic alterations.

## Introduction

Accumulation of DNA damage is a hallmark of genome instability and is associated with both aging and cancer [1-3]. Mice deficient in proteins involved in DNA damage sensing and repair exhibit severe deficiencies in these pathways leading to accelerated aging and oncogenic transformation [4]. Many progeria (premature aging) syndromes in humans are caused by mutations in genes encoding  proteins involved in  DNA repair and are associated with increased incidence of cancer [5,6].

**Figure 1.**
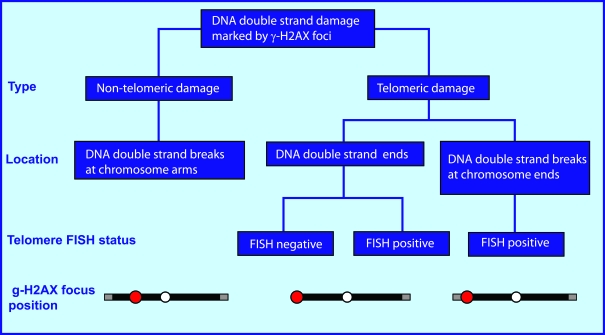
Types of endogenous DNA double-strand damage marked by γ-H2AX foci. The endogenous DNA double-strand damage that induces H2AX phosphorylation includes both non-telomeric DNA double-stand breaks (DSBs) located at chromatid arms and damaged telomeres. Telomeric damage is a chromosome end-specific damage which includes two types of lesions: 1) DNA double-strand ends which are generally the consequence of telomere dysfunction, though this type of damage can be also present at long telomeres when the telomere loop is open, and 2) DNA DSBs at telomeres.

One major type of DNA lesion leading genomic instability is DNA double-strand damage, which includes both telomere-independent DNA double-stand breaks (DSBs) and damaged telomeres (see schematic in Figure 1). Telomere-independent DSBs, which localize at chromosome arms, can be induced by a variety of agents including ionizing radiation, radiomimetic drugs, reactive oxygen species, metabolic errors during replication and transcription, and deficient DNA repair [7]. Telomeric damage is chromosome end-specific and includes two types of lesions, DNA double-strand ends which are the consequence of telomere dysfunction, and DNA DSBs at telomeres.

Immediately upon DNA double-strand damage formation, hundreds of histone H2AX molecules are phosphorylated at the break site to form γ-H2AX foci. This characteristic makes γ-H2AX foci a sensitive marker for DSB damage. An important finding, made possible by the use of antibodies to γ-H2AX, is that cells that have not been subjected to deliberate damage still contain endogenous DSB damage. This endogenous DNA DSB damage is present at low levels in early passage primary cells, but it increases in human and mouse cells during *in vivo* aging and *in vitro* cellular senescence [3,8,9]. Increased and variable levels of DNA DSBs have also been found in premalignant lesions, tumor cell lines and tumors of different origins [2,10-13]. The endogenous γ-H2AX foci contain DNA DSB repair factors such as 53BP1, MRE11, RAD50, and NBS1, indicating that DNA DSB repair is being attempted at these sites [3,9].

The existence of non-telomeric DNA DSBs and telomeres-associated endogenous DNA double-strand damage creates confusion about which type of damage is present. The confusion can be clarified by determining the location of the γ-H2AX foci on the chromosomes. When this type of analysis was performed on human and mouse senescent cells, both were found to contain similar levels of total endogenous DNA DSB damage, but differing contributions from non-telomeric DSBs and damaged telomeres. This comparison of human and mouse cells suggested that both telomere-independent and telomere-associated damage may be similarly involved in the signaling to induce cellular senescence and organismal aging [14].

In the present study we performed this analysis on five tumor cell lines to clarify the relative contribution of telomeric damage to the high level of endogenous DNA damage in tumors. We report that the numbers of non-telomeric DNA DSBs, as measured by γ-H2AX foci present at chromosome arms, were remarkably similar across all cultures studied. However, the numbers of γ-H2AX foci associated with telomeres varied considerably and correlated inversely with telomerase activity. These results indicate that human tumor cells contain substantial and variable numbers of dysfunctional telomeres, which account for most of the variation in the number of γ-H2AX foci in different human tumor lines.

## Results

### Distribution of γ-H2AX foci in proliferating tumor cell cultures

**Figure 2.**
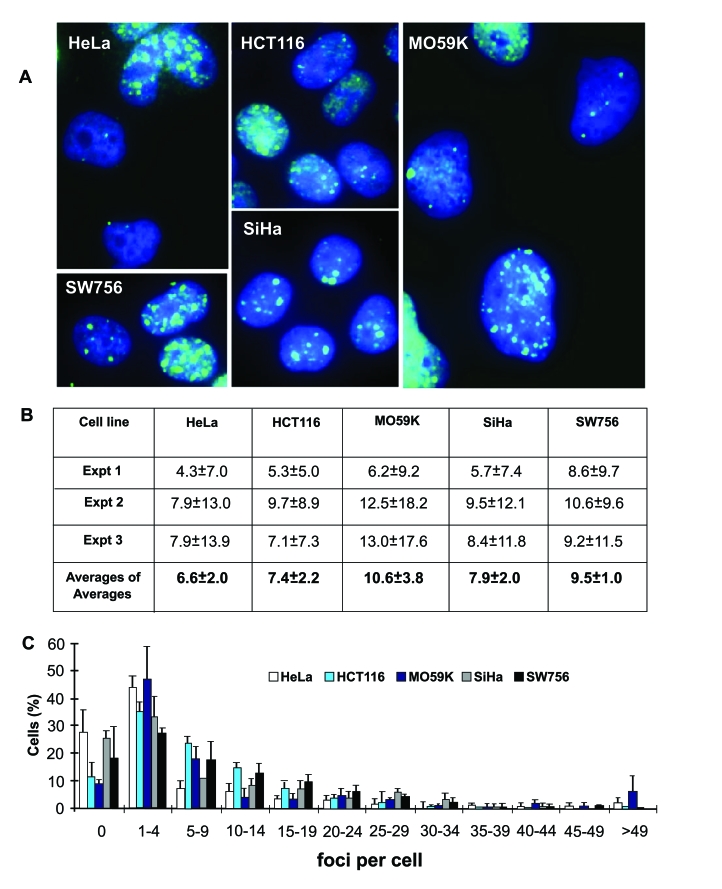
Endogenous γ-H2AX foci in interphase cells of five human tumor cell lines. **(A)** Images of endogenous γ-H2AX foci (green) in untreated HeLa, HCT116, M059K, SiHa and SW756 cells. DAPI staining (blue) indicates DNA. **(B)** Average numbers of γ-H2AX foci per cell in three independent experiments (Expt 1-3) with high SDs (n is at least 70 cells counted in each experiment), and average of averages from these experiments (n=3). **(C)** Fractions of cells in the five tumor cell populations with the noted numbers of γ-H2AX foci.

 In contrast to senescent cells, which contain similar numbers of endogenous γ-H2AX foci irrespective of origin [14], malignant cells have higher DSB levels which vary greatly in different cultures and tumors [10,12,13]. We performed parallel analyses of γ-H2AX foci in undamaged cultures of five tumor cell lines of different origins, HeLa, SiHa, and SW756 (cervical carcinomas); HCT116 (colon carcinoma) and M059K (glioblastoma) (Figure 2). Endogenous γ-H2AX levels in these cultures have been shown earlier to vary widely, from an average of 1.1 γ-H2AX foci per cell in M059K cells to as high as 46 foci per cell in SW756 cells [10,13]. Additionally, comparison of DNA damage in 6 intact cervical carcinoma cell lines showed great variability in γ-H2AX focal numbers, indicating that endogenous DNA damage is independent of tumor origin [10]. In this study we counted γ-H2AX foci in interphase in large cell populations (400 - 600 cells) of the five lines, and found an average of 6.6 -10.6 foci per cell (Figure 2A, B).  Cultures of the same tumor line yielded average numbers of γ-H2AX foci per cell that varied by over two-fold in three independent experiments, indicating that focal numbers are dependent on culture conditions (Figure 2B). In addition, in these three experiments, the standard deviations were often larger than the average values for the number of γ-H2AX foci per cell, indicating a large amount of heterogeneity in the population. The cause of these large standard deviations may be explained by data shown in Figure 2C. In each tumor line, while the majority of the cells contained less than 10 foci per cell, there was a substantial fraction of cells that contained larger numbers of γ-H2AX foci, up to about 50 per cell, creating a long tail in the distribution and leading to large standard deviations from the average.

The counts we present here are different from published data for these cell lines. We account for this discrepancy by possible bias caused by a great disparity in the number of γ-H2AX foci in a cell population, in the focal sizes and intensities (Figure 2A), and by variations in the cells' proliferative status, as well as their checkpoint status and expression of p53 or other proteins involved in genomic stability that could have changed due to genetic drift over time. Therefore, since counting γ-H2AX  foci  in interphase tumor cells can provide only limited information, studies in metaphase cells were performed to allow visualization of truly informative foci by avoiding at least some of these problems, such as proliferative status and focal variability.

### Origins of endogenous γ-H2AX foci in metaphase tumor cells

**Figure 3.**
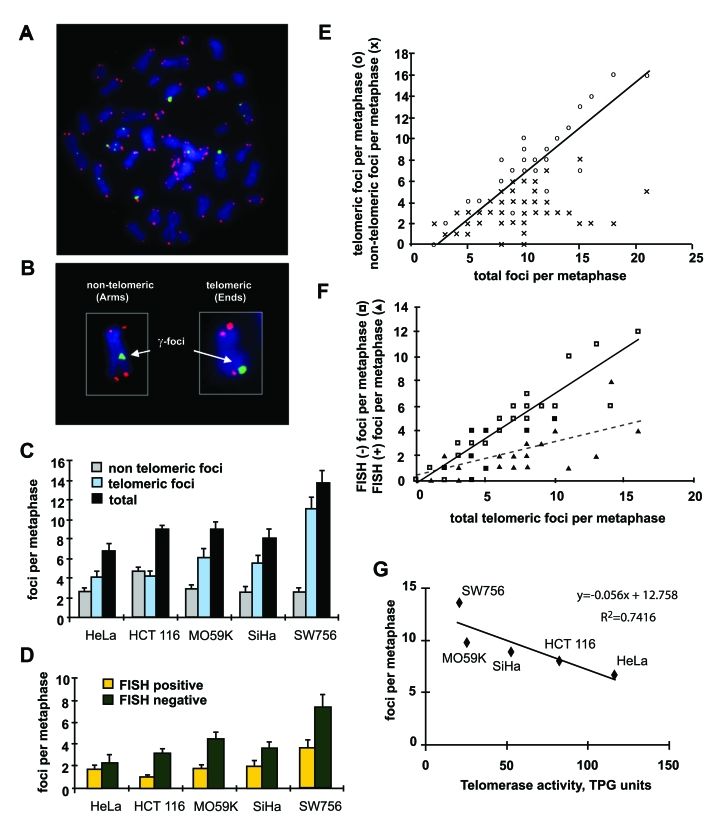
Distribution of γ-H2AX foci on metaphases of human tumor cells. **(A)** Metaphase spread of HCT116 cells stained for γ-H2AX (green) and telomeric DNA (red). **(B)** Scoring of γ-H2AX foci as along chromatid arms (Arms) or on chromatid ends (Ends). **(C)** The numbers of γ-H2AX foci in metaphases from five tumor cell lines as noted. Foci are noted as non-telomeric (Arms, gray), telomeric (Ends, blue), and total (black). **(D)** Telomeric γ-H2AX foci with (yellow) and without (green) telomere FISH signal in the five tumor lines. At least 10 metaphases were screened per data point in independent experiments. Error bars signify standard errors. **(E) **Numbers of telomeric (open circles) and non-telomeric (cross hatches) foci vs. the total numbers of γ-H2AX foci on the metaphase spreads of the five tumor cell lines. The data from all five tumor cell lines was pooled for this analysis. **(F) **Numbers of FISH negative (open squares) and FISH positive telomeric (filled triangle) γ-H2AX foci vs. total telomeric foci in all checked metaphases of the five tumor cell lines. **(G)** Reverse correlation of the numbers of γ-H2AX foci and telomerase activity in the five tumor cell lines. TPG is a total product generated corresponding to 600 molecules of telomerase substrate primers extended with at least four telomere repeats [28].

Yu et al. reported that tumor cell cultures exhibited large numbers of endogenous γ-H2AX foci per cell, sometimes equivalent to several Gy of ionizing radiation. Strikingly however, they found no difference in  tail  moments  when  these cultures were identically irradiated and the cells were analyzed by the comet assay [12]. This discrepancy suggests the hypothesis that a substantial fraction of the endogenous γ-H2AX foci might be marking uncapped telomeres rather than DSBs. Since the damage is at the end of the DNA, the comet or any other DNA fragmentation assay would not detect it. To examine this notion, we analyzed metaphases of five tumor cell lines for γ-H2AX and telomeric DNA FISH signals to score the numbers of telomere-associated and telomere-independent  γ-H2AX foci (Figure 3). This procedure permits the localization of γ-H2AX foci to either the chromatid arms, corresponding to DNA DSBs of non-telomeric origin, or to the ends of the chromatids, corresponding to either DSB-damaged telomeres (FISH-positive terminal foci), or double-strand ends at critically short telomeres lacking detectable telomere repeats (FISH-negative foci) (Figure 3A, B).

When the distribution of γ-H2AX foci on metaphase spreads was analyzed, the total numbers per cell varied similarly to the average number of foci found in the interphase nuclei (Figure 3C, black bars). Strikingly, the numbers of γ-H2AX foci along the chromosome arms were found to be similar in all cell lines (Figure 3C, gray bars). In fact, in four of the cell lines the numbers were the same within the standard error, with an average of 2.6 foci per cell. Only HCT116 exhibited a different number of γ-H2AX foci on chromatid arms, 4.7 per cell. These results suggest that the number of DNA DSBs may have fairly constant values among tumor lines. In contrast, the numbers of telomeric γ-H2AX  foci  were  more  variable  among the five lines (Figure 3C, blue bars), suggesting that the differences in endogenous γ-H2AX focal numbers are primarily due to variations in the number of damaged telomeres. When the damaged  telomeres containing  γ-H2AX foci  were classified as to whether they were FISH positive or negative, the majority were found to be FISH negative, confirming that telomeres were critically short (Figure 3D).

We next analyzed the metaphase spread data to discern the distribution of telomeric and non-telomeric γ-H2AX foci in the cells with increasing numbers of total foci (Figure 3E). This analysis demonstrates that in cells that contain more than the average number of γ-H2AX foci, the increase is almost completely due to telomeric foci. This result indicates that tumor cells maintain a fairy constant level of non-telomeric DNA DSBs irrespective of the total DNA damage, and it is damaged telomeres that become more plentiful in these cells. Similar analysis of the distribution of FISH-negative and FISH-positive telomeric γ-H2AX foci indicates that among the total telomeric foci per metaphase, critically short telomeres account for disparities (Figure 3F).

A defining characteristic of cancer cells is the presence of telomerase, which permits these cells to divide indefinitely [15,16]. Since telomeres are maintained by telomerase, which catalyzes the addition of telomeric DNA repeats to the chromosome ends [17,18], we asked whether the average telomerase activity correlated with the average numbers of γ-H2AX foci in the five studied tumor lines. We found an inverse relationship between the numbers of γ-H2AX foci and telomerase activity (Figure 3G). These results indicate that the level of telomerase in a tumor cell line is a major determinant of the average number of γ-H2AX foci.

## Discussion

The purpose of this study was to determine how much of the DNA double-strand damage in tumor cells is actually due to damaged telomeres. The results clearly show that damaged telomeres make up the majority of DNA double-strand damage in tumor cells, and that cells with more foci contained more damaged telomeres, while the numbers of telomere-independent DSBs remained fairly constant throughout the population. The numbers of endogenous telomeric γ-H2AX foci in metaphases correlated inversely with telomerase activity in these cell lines, confirming the importance of telomerase in malignant phenotypes. These data parallel our recently published findings for senescent cells which also contain elevated γ-H2AX foci compared to actively growing low population doubling cultures, which in humans have mainly telomere-associated origins [14]. Telomere shortening and consequent telomere dysfunction or uncapping are associated with many human diseases including aging and cancer, and have received a great deal of attention (reviewed in [19,20]). Genomic alterations observed in human cancers can be caused by inappropriate DNA repair taking place at dysfunctional telomeres leading to loss of heterozygosity, chromosomal rearrangements, aneuploidy, and repression of DNA damage checkpoints [21].  Shorter telomeres have been associated with increased cancer risk [22]. Differences in telomere-associated DNA damage in different tumor cell lines can be explained partly by the fact that these cell lines have been derived from different individuals, thus telomere lengths are affected by the cellular activity of telomerase, the cells' history of cell division and environmental factors. Additionally, as the tumor lines were isolated many years ago, they may have changed due to genetic drift. Finally, telomere length is tissue-specific, and age-dependent [23,24], and there is considerable heterogeneity between humans [25].

Telomerase expression is one of the most clearly distinguishable characteristics between malignant and primary healthy cells [15] which makes it a suitable target for cancer therapy. Inhibiting telomerase activity in tumor cells may increase the number of damaged telomeres and thereby limit proliferation. Many telomerase inhibitors are now going through clinical trials [26]. However, previously there was no tool to analyze whether tumors show different sensitivity for telomerase inhibitors and to control this sensitivity. Here we show that each tumor cell line has a signature amount of telomere-associated DNA damage. Therefore, telomerase inhibitors or telomere maintenance-targeting drugs could affect different tumors with differing success, and analysis of telomere-associated γ-H2AX focal numbers in primary tumors treated with telomerase-based drugs could be used to monitor the drug efficiency. In addition, many cancer drugs act by introducing sufficient excess DNA damage into a tumor cell to prevent further proliferation. The procedure presented here enables researchers to determine the extent of the two types of DNA double-strand damage, both of which are relevant to cancer treatment, and provides useful information for developing tailor-made cancer therapy.

## Methods

Cell cultures. HeLa, SiHa and SW756 (cervical carcinomas), HCT116 (colon carcinoma), and M059K (glioblastoma) cell lines were obtained from ATCC (Manassas, VA) and grown in D-MEM medium containing 10% fetal bovine serum. Cells were maintained in a humidified incubator at 37ºC, 5% CO_2_ and 20% O_2_.

Immunocytochemistry. Cell cultures were plated on Labtek II slides (Nalge Nunc International, Naperville, IL). After the cultures reached 80% confluency, they were fixed with 2% paraformaldehyde for 20 min. Then the cells were washed 4 times with PBS, permeabilized with pre-chilled 70% ethanol at -20ºC and stored overnight at 4ºC. PBS was replaced with PBS containing 0.5% Tween-20 and 0.1% Triton X-100 (Bio-Rad Laboratories, Hercules, CA) for blocking and antibody incubations. The samples were stained with primary mouse monoclonal anti-γ-H2AX antibody (Abcam Inc., Cambridge, MA) followed by secondary Alexa-488-conjugated anti-mouse IgG (Molecular Probes, Eugene, OR). Nuclei were counterstained with DAPI (4,6-diamidino-2-phenylindole-dihydrochroride). Images were acquired with the BD Pathway Bioimager and processed with Attovision software (Becton Dickinson Biosciences, San LoseJose, CA). γ-H2AX foci were counted by eye in three independent experiments, in a total of 400-600 cell nuclei.

Immunocytochemistry and FISH. Metaphase spreads were prepared as described previously [27]. The slides were stained with mouse monoclonal anti-γ-H2AX antibody followed by Alexa-488-conjugated anti-mouse IgG. The staining with both γ-H2AX and telomere FISH was performed according to the telomere FISH kit (DakoCytomation, Glostrup, Denmark) protocol with some modifications. Briefly, the γ-H2AX stained cells were fixed with 50 mM ethylene glycol-bis (succinic acid N-hydroxy-succinimide ester) (Sigma, St. Louis, MO). The hybridization was performed according to the kit protocol. DAPI was used for visualization of DNA. The signal was detected with Olympus fluorescent microscope (Olympus America Inc. Melville, NY).

Telomerase assay *.* Telomerase activity in tumor cell lines was analyzed using the TRAPeze Telomerase Detection Kit (Chemicon International a division of Serologicals Co., Temecula, CA). Cell extracts, prepared according to the manufacturer's instructions, were assayed for telomerase activity in 50 μL reactions provided with the TRAPeze Telomerase Detection Kit with the exception of Platinum Taq DNA polymerase (Invitrogen, Eugene, OR). The reaction mixtures were size-fractionated by electrophoresis in a 10% non-denaturating polyacrylamide gel and stained with SYBR Green 1 dye (Sigma). The gels were photographed using the Typhoon 8600 system (Amersham Pharmacia Biotechnology, Piscataway,   NJ).
